# Restoring Retinal Function in a Mouse Model of Hereditary Blindness

**DOI:** 10.1371/journal.pmed.0020399

**Published:** 2005-11-29

**Authors:** Tony Moore

## Abstract

Moore discusses a new study showing rescue of photoreceptor function using gene and drug therapies in a mouse model of Leber congenital amaurosis.

Leber congenital amaurosis (LCA) is a generic term used to describe a heterogeneous group of inherited disorders that result in severe loss of rod and cone photoreceptor function in infancy and early childhood. Although each individual disorder is rare, LCA accounts for 3%–5% of childhood blindness [1].

To date, ten different genes have been implicated in such early onset retinal dystrophies. The genes are expressed in the photoreceptors or the underlying retinal pigment epithelium (RPE), and encode a variety of proteins, including structural proteins, transcription factors, and key components of the phototransduction cascade and the visual cycle.

## The Visual Cycle

**Figure 1:**
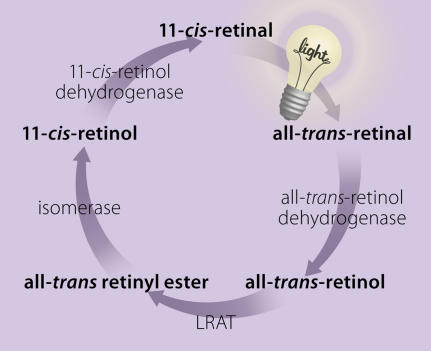
The Visual Cycle (Illustration: Giovanni Maki, adapted from [4])

Light perception in vertebrates is mediated by a group of G protein–coupled receptors called opsins, which are bound to a chromophore (light-absorbing compound), 11-*cis*-retinal, derived from vitamin A. Absorption of light induces an 11-*cis* to all-*trans* isomerisation of the chromophore, and the ensuing conformational change initiates the visual transduction cascade. In order for the activated opsin to participate again in the visual process, the all-*trans*-retinal must be isomerised back to the 11-*cis* form via an enzymatic pathway, the visual cycle (Figure 1). This occurs mainly within the RPE.

Genes encoding a number of enzymes within the visual pathway have been implicated in LCA, namely, *RPE65* (retinoid isomerase), *retinal dehydrogenase 12*, and *lecithin:retinol acyl transferase (LRAT)*. The genetic mutations lead to a lack of 11-*cis*-retinal, resulting in severe loss of photoreceptor function and ultimately in cell death. Therapeutic approaches aimed at restoring levels of 11-*cis*-retinal by either improving enzyme function or using other biochemical strategies hold promise for restoring visual function. The development of good animal models of LCA is an essential first step in developing effective therapies.

## Promising Treatments in a Mouse Model

Batten et al., in this issue of *PLoS Medicine*, report very encouraging results of two different therapeutic approaches in the *Lrat* knockout *(Lrat^−/−^)* mouse [2]. The mutant mice show an early onset rod–cone dystrophy and, as in the human disorder, have disease confined to the eye. The mouse model appears to faithfully reproduce the phenotype seen in humans with *Lrat* deficiency. In the first approach, the authors used gene therapy with subretinal injection of a recombinant adeno-associated virus carrying the *Lrat* gene. Treated animals showed high expression of *Lrat* in the RPE adjacent to the injection site, markedly increased levels of visual pigment, and improvement of rod photoreceptor function measured electrophysiologically. The rescue of retinal function peaked at six weeks and then slowly declined. The reasons for the lack of sustained rescue are unclear.

A second approach was to use the orally administered pro-drugs 9-*cis*-retinyl acetate and 9-*cis*-retinyl succinate to bypass the Lrat stage of the visual cycle. (9-*cis*-retinoids were chosen as they are easier to synthesise and are more stable than 11-*cis*-forms.) Again, rescue of photoreceptor function could be unequivocally demonstrated. Furthermore, treatment with both gene therapy and synthetic retinoids was more effective than treatment with either alone.

## Clinical Implications

What is the relevance of these results for patients with early onset retinal dystrophies and their clinicians? Clearly, there is cause for optimism in that treatment may become available within the next few years. Gene therapy is perhaps the most promising approach. Photoreceptor rescue has now been demonstrated in a number of animal models of inherited retinal disease, including mice and dogs that lack RPE65 [3]. The first gene therapy trial in humans with early onset retinal dystrophies as a result of mutations in *RPE65* is due to start within the next two years, and if this is successful, treatment of other disorders such as *Lrat* deficiency will follow. However, unlike *RPE65*, there is no large animal model of *Lrat* deficiency, and patients with *Lrat* deficiency are extremely rare; these two problems may limit the development of a treatment trial in humans. To date, only three patients with *Lrat* deficiency have been described, and there is very limited information about the clinical phenotype and natural history.

What about dietary treatment? This has been used with limited success in other rare retinal dystrophies in humans, for example, Refsum disease and gyrate atrophy, where the biochemistry of the disorder is understood. The results of Batten and colleagues' study suggest that *Lrat* deficiency may also respond to dietary treatment [2]. It is very interesting that 9-*cis*-retinoids can replace 11-*cis*-retinal, and partially restore photoreceptor function. However, as the authors caution, there is much more work that needs to be done before such compounds can be used in humans. We will need to know whether 9-*cis*-retinoids are well tolerated in humans, whether there is any retinal or systemic toxicity, and whether retinal function is improved long term with prevention of cell death. Will the treatment be effective relatively late in the disease process when most patients tend to be diagnosed, or is early treatment essential?

## The Next Steps

Batten and colleagues' excellent study highlights the improvements that are being made in our understanding of the disease mechanisms in inherited retinal disease [2]. This knowledge will lead to the development of novel therapies for specific forms of disease. The challenge for clinicians is to be prepared for treatment trials. We need to identify the genetic mutations causing retinal degeneration in our patients, carefully characterise the phenotype and natural history of disease, and identify those patients who may benefit from the new therapeutic approaches.

## Abbreviations

LCA: Leber congenital amaurosis
LRAT: lecithin:retinol acyl transferase
RPE: retinal pigment epithelium
